# The efficacy and safety of Banxia-Houpo-Tang for chronic pharyngitis: A protocol for systematic review and meta analysis

**DOI:** 10.1097/MD.0000000000019922

**Published:** 2020-07-24

**Authors:** Chenyi Xu, Rensong Yue, Xuelian Lv, Tingchao Wu, Maoyi Yang, Yuan Chen

**Affiliations:** a Hospital of Chengdu University of Traditional Chinese Medicine; b Hospital of Traditional Chinese Medicine of Xinjin, Chengdu, P.R. China.

**Keywords:** Banxia-Houpo-Tang, Chinese herbal medicine, chronic pharyngitis, protocol

## Abstract

**Background:**

Chronic pharyngitis is a common disease with a dry throat, sore throat, pharyngeal itching, dry cough, and difficulty in swallowing, bringing inconvenience to patients’ daily life. Banxia-Houpo-Tang (BHT) has proven to be effective in the treatment of chronic pharyngitis, yet its real extent is not well understood. To prove this point, we will perform a protocol for a systematic review and meta-analysis of BHT for chronic pharyngitis.

**Methods/design:**

We will search for electronic databases both English and Chinese from inception to December 2019. Two experienced researchers select the qualified articles from: The Cochrane Library, EBM Reviews, OVID, Web of Science, PubMed, Chinese National Knowledge Infrastructure (CNKI), China Academic Journal Network Publishing Database (CAJD), China Biomedical Literature database (CBM), VIP Database for Chinese Technical Periodicals (VIP). Journal Integration Platform and WAN FANG Database. We select the appropriate searching language. The primary outcome was remission rate, and the secondary outcomes include clinical symptoms, clinical examination, adverse event. Data extraction and quality assessment will be conducted by 2 experienced researchers independently. Data analysis and the risk of bias assessment will be determined by RevMan 5.3 software.

**Results:**

Based on the current proofs, we will get the exact evidence about the safety and effectiveness of BHT in the treatment of chronic pharyngitis.

**Conclusion:**

Our study is the first meta-analysis to evaluate the efficacy and safety of BHT in the treatment of chronic pharyngitis, and it will provide evidence for alternative treatment for the management of chronic pharyngitis.

**OSF Registration number:**

DOI 10.17605/OSF.IO/QNF6X

## 1 Introduction

Chronic pharyngitis is a common otolaryngology disease mainly caused by infectious etiologies such as viral, bacteria,^[1–2]^ or other factors for a lifestyle like alcohol abuse, overuse of voice. Non-infectious diseases such as gastroesophageal reflux and recurrent acute pharyngitis^[3]^ also need to be considered. The main clinical manifestations of this disease include recurrent hoarseness, irritating cough, sore throat, and difficulty in swallowing. Sore throat, most of which are caused by a viral infection, lead to 5% children and 2% outpatient visited in clinical activities,^[4–7]^ and puts enormous economic pressure on patients. Over 13 million visit the doctor annually in the United States related to this disease.^[8,9]^ In the UK, the loss of productivity and doctors’ office visits due to pharyngeal pian was £400 million a year.^[10,11]^

At present, growing evidence reveals that antibiotic therapy and adenotonsillectomy surgical therapy are both effective measures in the treatment of the disease.^[12]^ Some chronic diseases with pharyngitis like tonsillitis threaten the health of patients especially children.^[12]^ In many countries, surgery of the tonsils is still the main treatment therapy for tonsillitis. However, because of the change of the disease indications and lack of evidence-based study, surgery for this disease needs further evidence.^[13,14]^ And severe sore throat may still trouble patients after tonsillectomy.^[15]^ Although streptococcus pyogenes or group A β-haemolytic streptococcus (GABHS) is the major causative agent of acute pharyngitis, broad-spectrum antibiotics abusing without pathogen culture can be seen everywhere for pharyngitis.^[16]^ Pharyngitis was one of the diseases that account for one-third of all antibiotics prescribed in outpatient settings in American.^[17]^ But, the value of antibiotics is in reducing complications rather than treating them, so antibiotics are ineffective therapy in the treatment of sore throat, which (40%–80%) caused by viral infections.^[13]^ Recently studies indicate that insufficient evidence for antibiotics prevents sore throat.^[3]^ Antibiotic treatment is controversial in some high-income countries.^[18–21]^ So, neither surgery nor antibiotics are effective treatments for pharyngitis. A reasonable choice with fewer side effects and few cost treatments are receiving increasing attention.^[22]^

Some Chinese herbal medicine has been proven effective for treating sore throat,^[23]^ but as a result of the inadequate presentation of the evidence, many effective and natural therapies cannot be recommended. Banxia-Houpo-Tang (BHT) is a prescription with thousands of years of clinical experience in China, and it is still widely used nowadays.^[24–26]^ BHT is comprised of Banxia (*Pinellia ternata*), Houpo (*Mangnolia*), Fuling (*Poria cocos*), Shengjiang (Fresh ginger), Suye (Perilla leaf). However, no systematic review and meta-analysis aim to explain its efficacy and safety. To find natural medicine and further study, we provide a protocol to evaluate the safety and effectiveness of BHT for chronic pharyngitis.

## 2 Objectives

To establish a systematic and comprehensive approach for locating the evidence, a review and meta-analysis will be used to whether the Banxia-Houpo-Tang is effective or can it ease the pressure of antibiotic abusing in the treatment of pharyngitis. Risk assessment and identify the most promising measures.

## 3 Methods and analysis

### 3.1 Registration and review design

This study have been registered on the Open Science Framework (OSF) platform and the OSF registration number is DOI 10.17605/OSF.IO/QNF6X. The research process will be compliant with the Preferred Reporting Items for Systematic Review and Meta-analysis Protocols (PRISMA-P)^[27]^ guidelines.

### 3.2 Search strategy

We will search for electronic databases both English and Chinese from inception to December 2019. Two experienced researchers select the qualified articles from: The Cochrane Library, EBM Reviews, OVID, Web of Science, PubMed, Chinese National Knowledge Infrastructure (CNKI), China Academic Journal Network Publishing Database (CAJD), China Biomedical Literature database (CBM), VIP Database for Chinese Technical Periodicals (VIP). Journal Integration Platform and WAN FANG Database. Endnote 9.0 will be used to manage literature, export references, and pick articles after group discussion. Medical subject headings (Mesh) or (and) random words will be selected in electronic databases: Banxia-Houpo-Tang, Chinese herbal medicine, chronic pharyngitis, sore throat, dry throat, pharyngeal itching, dry cough, difficulty in swallowing. The literature search process of PubMed is performed in Table 1.

**Table 1:**
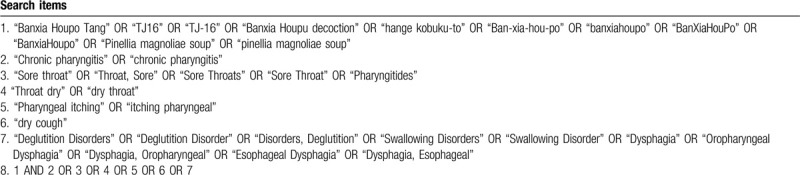
Example for PubMed search strategy.

### 3.3 Inclusion and exclusion criteria

#### 3.3.1 Study design

All the clinic trials of prospective, randomized, multi-center, double-blind, parallel-group, placebo-control studies about BHT (or Chinese patent medicine for BHT) will be included. Clinical observations without randomized, or placebo-control or follow-up will not be included.

#### 3.3.2 Participants

Clinical diagnosis of chronic pharyngitis by the Otolaryngologist. According to the patient's history of continuous pharyngeal discomfort for >3 months, combined with the patient's pharyngeal mucosa chronic congestion. Specific diagnostic criteria^[28]^ are as follow (Table 2).

**Table 2:**
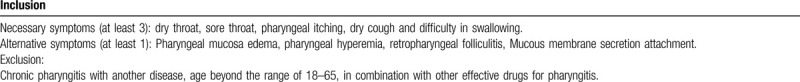
Inclusion and exclusion.

#### 3.3.3 Interventions

The intervention in the treatment group is BHT (Has passed the national department concerned certification of proprietary Chinese patent medicine or granules or decoction). The intervention in the control group included placebo or medicines that have proven effective at preventing chronic pharyngitis.

#### 3.3.4 Outcomes

The primary outcome was remission rate. Remission was defined as all the uncomfortable symptoms such as dry throat, sore throat, pharyngeal itching, dry cough, difficulty in swallowing are not recurrence in 6 months. And pharyngeal mucosa without swollen, atrophic, hyperemic, and follicular hyperplasia in clinical signs. Secondary outcomes included: the recurrence rate (1 month, 3 months, 6 months) after treatment, remission rates of symptoms and signs (the ratio of the total score before treatment minus the total score after treatment and the total score before treatment), evaluation of adverse events.

### 3.4 Study selection

**Figure 1:**
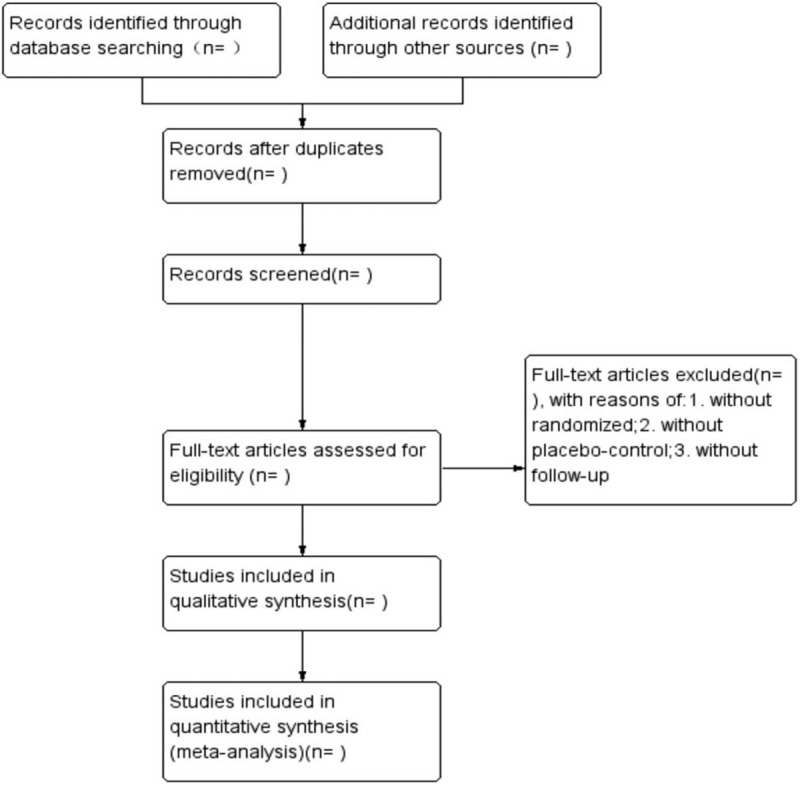
The flowchart of study selection.

Standardized training will be conduct before collecting the data from the electronic database above. The studies selected from the databases are integrated into Endnote X9 (Thomson Reuters https://www.endnote.com/). Two reviewers will screen the titles and abstracts of all studies to identify potential articles according to inclusion and exclusion criteria. If there was any disagreement, we will have a group discussion. After group discussion, all the articles passed are reviewed by a third author (XL). A flowchart to show the whole process of study selection (Fig. 1).

### 3.5 Data extraction and missing data management

Two authors will fill in a standardized form by extract data individually. This collection form will include patient characteristics, study design, control intervention, result measures, and effects. According to Cochrane Handbook for Systematic Reviews of Interventions for inclusion characteristics study,^[29]^ the specific requirements are as follows: The first author's name, the year of publication, nationality of the author, the number of included cases, intervention and control measures, disease duration, period of observation and follow-up, symptoms and signs of outcomes, routine examination of safety, age, and sex of patients. The missing data will be retrieved by contact with the author.

### 3.6 Assessment of study quality and risk of bias

According to the Cochrane Handbook for Systematic Reviews of Interventions,^[29]^ 2 investigators will evaluate each of the included randomized controlled trial (RCT), including the application of hidden, blind methods, information data for the generation, distribution of randomized control sequences, selective reports, and other possible problems. The risk of bias will be marked as high, uncertain and low: low risk of bias is defined as “existence bias does not affect the results of the study,” the risk of uncertainty bias is defined as “the bias of existence raises doubts about the results of the study,” the high risk of bias is defined as “the bias of existence seriously affects the credibility of the results.” Two investigators will use the Grading of Recommendations Assessment Development and Evaluation (GRADE) system^[30]^ for evidence evaluation. If there any questions, a third participator assessment will be used.

### 3.7 Data synthesis and analysis

#### 3.7.1 Measures of treatment effect

Data statistics analysis will be performed using Review Manager 5.3 software (Cochrane Collaboration, Denmark) and Stata 14.0 (Stata Statistical Software: Release 14. College Station, TX: Stata Corp LP). For dichotomous variables: adverse events, we will use the Relative Ratio (RR) value with 95% confidence intervals (CI) of the comparison, effective or not, will be described by using the OR value with 95% CI. For continuous variables, a combined statistical analysis will be performed by mean difference (SD) and standardized mean difference (SMD) with 95% CI.

#### 3.7.2 Assessment of heterogeneity

Heterogeneity analysis was used to test whether the results of each independent study were combinable or not. In this study, the *I*^2^ statistic will be figuring out to evaluate the results of the included studies. A high level of heterogeneity was referred to *I*^2^ ≥ 50, the random-effects model was recommended. A low level of heterogeneity was referred to *I*^2^ < 50, the fixed effects model was suggested.

#### 3.7.3 Publication bias

If >10 standard-compliant studies are included, a funnel chart will be adopted to evaluate publication bias reports. If there is no publication bias, the data from these studies will present an inverted funnel shape and be symmetrically distributed. If an asymmetrical inverted funnel plot appears, the bias of the study samples will be presented. Egg and Begger tests will be used to detect the asymmetry of the funnel plot.

#### 3.7.4 Subgroup analysis

Subgroup analysis is used to find the reasons for heterogeneity. We will use the secondary data of the studies to build a meta-review model, screen out the influencing factors of heterogeneity, and conduct subgroup analysis for these influencing factors (such as sex, age, or subgroups of the disease).

#### 3.7.5 Sensitivity analysis

Sensitivity analysis is used to analyze the stability of the results, to exclude the study of abnormal results (such as samples with too large or too small values), and then to re-examine the meta-analysis to found if there is any change in the conclusions. Every time a study is excluded, analyze the stability of the results. If there is no essential difference between the results before and after the sensitivity analysis, it reveals that the results of this Meta-analysis were stable.

### 3.8 Ethical review

Our research without directly relates to individual patients and therefore the issue of ethical review does not exist.

## 4 Discussion

Currently, many patients are plagued by chronic pharyngitis, so drug abuse is widespread all over the world. To date, the attention of chronic pharyngitis is still insufficient, and there is not even a systematic evaluation of drug treatment for chronic pharyngitis. Traditional Chinese medicine resource is a treasure house that contains many effective methods that have not been proven by modern science. The purpose of this evaluation is to find safety and effective method for the treatment of chronic pharyngitis through existing published articles and provide some useful data support for the treatment of chronic pharyngitis and the development of later drugs. Therefore, we will systematically evaluate this study accordance with the requirements of the Cochrane Handbook for Systematic Reviews of Interventions^[29]^ strictly, and the results will be published in peer-reviewed publications to provide a reference for the treatment of this disease.

### 4.1 Study funds

This project is funded by the National Natural Science Foundation of China (No. 81774279) and the Key Research and Development Project of Sichuan Province, Science and Technology Department of Sichuan (No. 2018SZ0068). The sponsors are not involved in design, execution, or writing the study.

## Author contributions

**Conceptualization:** Chenyi Xu, Rensong Yue.

**Methodology:** Chenyi Xu, Rensong Yue.

**Supervision:** Xuelian Lv, Yuan Chen, Tingchao Wu.

**Writing – original draft**: Chenyi Xu, Xuelian Lv.

**Writing-review & editing:** Yang Maoyi, Tingchao Wu.
